# Transfusion-Related Cost and Time Burden Offsets in Patients with Myelofibrosis Treated with Momelotinib in the SIMPLIFY-1 and SIMPLIFY-2 Trials

**DOI:** 10.3390/cancers16234067

**Published:** 2024-12-05

**Authors:** Lucia Masarova, Tom Liu, Mirko Fillbrunn, Weilong Li, Gautam Sajeev, Sumati Rao, Boris Gorsh, James Signorovitch

**Affiliations:** 1 Department of Leukemia, Division of Cancer Medicine, The University of Texas MD Anderson Cancer Center, Houston, TX 77030, USA; 2 GSK plc, Philadelphia, PA 19104, USA; tom.x.liu@gsk.com (T.L.); sumati.a.rao@gsk.com (S.R.); bgorsh@gmail.com (B.G.); 3 Analysis Group, Inc., Boston, MA 02199, USA; mirko.fillbrunn@analysisgroup.com (M.F.); gautam.sajeev@analysisgroup.com (G.S.); james.signorovitch@analysisgroup.com (J.S.); 4 Analysis Group, Inc., New York, NY 10036, USA; weilong.li@analysisgroup.com

**Keywords:** ACVR1, anemia, best available therapy, cost, JAK inhibitor, momelotinib, myelofibrosis, ruxolitinib, time burden, transfusion

## Abstract

**Background/Objectives:** Red blood cell transfusions for anemia impose high financial and healthcare resource utilization burdens on patients with myelofibrosis (MF). This study estimates projected differences in medical costs and transfusion-related cost and time burdens with momelotinib vs. ruxolitinib or best available therapy (BAT) in Janus kinase (JAK) inhibitor–naive and –experienced patients. **Methods:** Analyses used 24-week transfusion data from the phase 3 SIMPLIFY-1 and SIMPLIFY-2 trials and cost estimates from a study of adult patients with MF using the US IBM MarketScan Commercial database. The analyses were stratified by transfusion status at baseline (transfusion dependent [TD], transfusion independent/requiring [TI/TR]). Subgroup analyses were conducted among patients with anemia (moderate anemia, hemoglobin ≥ 8 to <10 g/dL; moderate-to-severe anemia, hemoglobin < 10 g/dL) and for patients aged ≥65 years. Cost estimates for patients aged ≥65 years were extracted from a study using the Medicare Fee-for-Service database. **Results:** In JAK inhibitor-naive patients, momelotinib was projected to result in cost and time savings vs. ruxolitinib in both TD and TI/TR patients across all populations evaluated. Projected cost and time savings were also observed with momelotinib vs. BAT in JAK inhibitor-experienced patients across all populations evaluated, primarily in TD patients. **Conclusions:** These results suggest that momelotinib may provide medical and transfusion-related cost and time burden offsets for both JAK inhibitor-naive and -experienced patients with MF.

## Simple Summary

Myelofibrosis is a rare type of blood cancer that is characterized by a buildup of scar tissue in the bone marrow, increased spleen size, and symptoms including tiredness, night sweats, itching, and pain. More than one-third of patients diagnosed with myelofibrosis also have anemia, a condition in which the patient has a low red blood cell count and requires regular blood transfusions. The phase 3 SIMPLIFY-1 and SIMPLIFY-2 clinical trials showed that momelotinib reduced symptoms, spleen size, and the need for transfusions among patients with myelofibrosis. This reduction in the number of transfusions with momelotinib may lower costs and save time on transfusion-related activities such as procedures, travel, recovery, preparation, and waiting compared with the current usual treatment.

## 1. Introduction

Myelofibrosis (MF) is a rare hematologic malignancy that originates in hematopoietic stem cells, the clonal proliferation of which drives abnormal cytokine production and aberrant signaling of the Janus kinase (JAK)/signal transducer and activator of transcription (STAT) pathway [1]. MF is characterized by bone marrow fibrosis and chronic inflammation, and key manifestations of the disease include anemia, splenomegaly, and symptoms such as fever, night sweats, weight loss, and fatigue, which reduce patients’ overall quality of life (QOL) [1,2,3].

Anemia is a hallmark of MF and is the result of a complex and multifactorial process that includes upregulated expression of inflammatory cytokines, which in turn drive hyperactivation of activin A receptor type 1 (ACVR1). This results in decreased serum iron availability, followed by iron-restricted anemia [4]. Approximately one-third of patients with MF present with anemia at diagnosis (hemoglobin < 10 g/dL), with nearly all patients developing anemia over time [2,5]. Red blood cell (RBC) transfusions are the main treatment option for worsening or severe anemia in patients with MF [4]. RBC transfusion-dependent (TD) anemia is present in approximately one-quarter of patients at diagnosis, and nearly 50% of all patients with MF will require RBC transfusions within 1 year of diagnosis [2]. Anemia and transfusion dependence are among the most significant negative prognostic indicators of overall survival and QOL in patients with MF [4,6,7,8,9,10]. 

In addition to a negative impact on patients’ QOL, transfusion dependence imposes a high economic burden on patients, their caregivers, and the health system at large owing to considerable healthcare resource utilization (HCRU) and costs associated with transfusions and management of any accompanying complications [9,11,12]. In a retrospective analysis, transfusion dependence in patients with MF led to substantially higher rates of HCRU and total annual medical costs up to nine times higher compared with non-TD patients (USD 255,200 vs. USD 27,800, respectively), even after controlling for differences in age, sex, and baseline comorbidity [11]. 

While currently approved JAK inhibitors improve splenomegaly and constitutional symptoms, some may induce or worsen anemia, and management strategies for patients with anemia have limited effectiveness [4,5], leaving MF-related anemia as a crucial medical need [10]. Momelotinib is an oral JAK1/JAK2/ACVR1 inhibitor approved in September 2023 for the treatment of patients with MF and anemia and is designed to address the complex drivers of iron-restricted anemia and inflammation associated with MF [5,13]. A phase 2 translational biology study demonstrated that anemia-related benefits of momelotinib are linked to its inhibition of ACVR1, which decreases hepcidin and improves iron metabolism [14]. Momelotinib has demonstrated improvement in anemia and transfusion dependence, in addition to symptom and spleen responses, across three phase 3 trials, including SIMPLIFY-1 (NCT01969838) and SIMPLIFY-2 (NCT02101268) [15,16,17,18]. In both trials, although baseline transfusion independence/dependence (TI/TD) rates were comparable between treatment arms, more patients treated with momelotinib were TI and fewer were TD at week 24 vs. patients treated with the comparators (ruxolitinib or best available therapy [BAT], respectively) [15,16]. 

Given the reported clinical trial efficacy of momelotinib and its observed effects on transfusion dependence, treatment with momelotinib has the potential to reduce economic burden and HCRU relative to other treatments. The current analysis estimates the projected differences in costs of transfusion-related medical care and outpatient visits and time burden for patients with MF treated with momelotinib vs. ruxolitinib or BAT utilizing clinical trial data from SIMPLIFY-1 and SIMPLIFY-2.

## 2. Materials and Methods

### 2.1. Data Sources

Data on clinical efficacy of momelotinib relative to its respective comparator were extracted from SIMPLIFY-1 (N = 432) and SIMPLIFY-2 (N = 156). SIMPLIFY-1 was a randomized, double-blind, phase 3 trial of momelotinib vs. ruxolitinib. Eligible patients had International Prognostic Scoring System high-risk or intermediate-2–risk MF, or intermediate-1–risk MF associated with symptomatic splenomegaly, hepatomegaly, or anemia and/or unresponsiveness to available non–JAK inhibitor therapy. Patients were randomly assigned 1:1 to receive momelotinib (200 mg once daily) or ruxolitinib (20 mg twice daily) [15]. SIMPLIFY-2 was an open-label superiority study in patients who were currently receiving ruxolitinib at the time of study or previously treated with ruxolitinib with a history of hematologic toxicity. Patients were randomized 2:1 to receive momelotinib (200 mg once daily) or BAT (88.5% of whom were treated with ruxolitinib) [16]. 

For estimates of the transfusion-related costs associated with treating patients with MF, 2 separate studies were accessed from the published literature. One study used the IBM MarketScan Commercial database, which summarized medical, pharmacy, and transfusion-specific costs of adult patients with MF [11]; a second study provided cost-of-care estimates among patients with MF aged ≥65 years using the Medicare Fee-for-Service database [12] (Supplemental Tables S1 and S2).

A previously published, web-based survey administered to 100 patients from multiple institutions identified via Cooley’s Anemia Foundation provided estimates for transfusion-related time burden. The patients who completed the survey had a physician diagnosis of β-thalassemia and underwent ≥ 1 RBC transfusion in the 6 months prior to taking the survey. Survey results provided estimates on the mean time spent for 1 RBC transfusion procedure, including the following time components: preparation, waiting room, waiting for blood, RBC transfusion procedure and recovery, and total round trip travel time [19,20] (Supplemental Table S3).

### 2.2. Variables: Outcomes and Inputs

Outcomes were calculated using transfusion status and rate data from patients in the respective trials and the cost and time burden estimates from the literature. The following 3 outcomes were stratified by baseline transfusion status (TD vs. TI/TR) and then compared between the momelotinib and ruxolitinib/BAT groups: projected total annual medical costs (excluding drug costs), projected annual outpatient transfusion visit costs, and projected annual transfusion-related time burden.

Transfusion status criteria from SIMPLIFY-1 and SIMPLIFY-2 (Supplemental Table S1) determined the proportion of patients who were TI, transfusion requiring (TR), and TD at baseline and at week 24 in each trial and treatment arm. The terminal TI rate at week 24 was based on a prespecified definition of no transfusions and all hemoglobin levels of ≥8 g/dL in the last 12 weeks before week 24. TD status was defined as ≥4 RBC units transfused or a hemoglobin level of <8 g/dL in the prior 8 weeks. TR patients were classified as those who did not meet the criteria for TD or TI.

Using these inputs, projected cost and time burden differences were calculated for the overall study population, patients aged ≥65 years, and anemia subgroups from SIMPLIFY-1 [21] and SIMPLIFY-2, whereby moderate anemia was defined as a hemoglobin level ≥ 8 to <10 g/dL [22] and moderate-to-severe anemia was defined as a hemoglobin level < 10 g/dL [23].

### 2.3. Statistical Analyses of Transfusion Status and Rates in SIMPLIFY-1 and SIMPLIFY-2

The proportion of patients who were TD, TI, TR, anemic, and aged ≥65 years was calculated using individual patient-level trial data for both studies. Data were extracted including only patients with known transfusion status at week 24. A separate analysis also evaluated patients based on a rolling TI rate defined as no transfusions and all hemoglobin levels of ≥8 g/dL during any 12-week period through week 24. Data for this rolling TI rate were extracted for the overall population and the moderate-to-severe anemia population.

**Figure 1:**
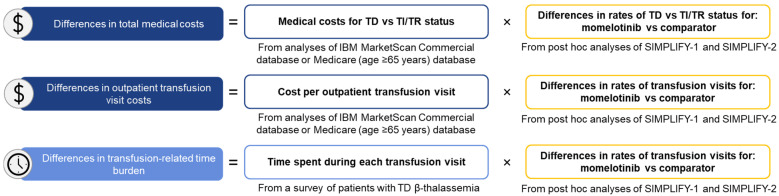
Method for estimating projected differences in transfusion-related cost and time burden for momelotinib vs. ruxolitinib (SIMPLIFY-1) and momelotinib vs. BAT (SIMPLIFY-2) ^a^. BAT, best available therapy; TD, transfusion dependent; TI, transfusion independent; TR, transfusion requiring. ^a^ Cost by transfusion status and per outpatient transfusion visit from Gerds A, et al. [12]. Time spent on each transfusion visit from Knoth RL, et al. [19].

The mean number of visits for RBC transfusions for the overall population, patients with anemia at baseline, and patients aged ≥65 years was calculated using individual patient-level data. Data were stratified by treatment arm and baseline transfusion status (TD or TI/TR, since the IBM MarketScan Commercial study only reported cost data for a combined TI/TR group). Rates of RBC transfusion visits observed over the 24-week trial period in each group were rescaled to reflect annualized rates (Figure 1).

### 2.4. Calculating Projected Differences in Cost and Time Burden

Projected medical costs for momelotinib vs. ruxolitinib or BAT were stratified by transfusion status per the criteria shown in Supplemental Table S1. For the overall population and anemic subpopulations of each trial, estimates of medical costs for momelotinib vs. ruxolitinib (SIMPLIFY-1) or momelotinib vs. BAT (SIMPLIFY-2) were calculated by multiplying the cost estimates from the IBM MarketScan Commercial study with the proportion of patients with TD or TI/TR status in each group at week 24. Cost estimates were calculated similarly for patients aged ≥65 years utilizing data from the Medicare Fee-for-Service database. Projected cost differences were calculated as the difference between the projected costs in the momelotinib treatment group and its respective comparator rounded to the nearest whole dollar (Figure 1). 

Projected outpatient transfusion costs were calculated by multiplying cost estimates per outpatient transfusion visit extracted from the literature with rates of transfusion visits in the momelotinib vs. ruxolitinib or BAT treatment arms in SIMPLIFY-1 and SIMPLIFY-2. 

Projected transfusion-related time burden was calculated by multiplying the estimated average time spent per transfusion visit reported by Cooley’s Anemia Foundation [19] with the average number of transfusion visits per patient per year in the momelotinib vs. ruxolitinib or BAT treatment arms from SIMPLIFY-1 and SIMPLIFY-2, respectively. Projected time savings were calculated as the difference between the projected time burden estimates for momelotinib vs. ruxolitinib or BAT (Figure 1). 

## 3. Results

### 3.1. JAK Inhibitor Naive (SIMPLIFY-1)

#### 3.1.1. Transfusion Status and Rates

The overall JAK inhibitor-naive population in SIMPLIFY-1 included 215 patients in the momelotinib arm and 217 patients in the ruxolitinib arm. Most patients in both arms retained their baseline transfusion status at week 24, although more patients were TD with ruxolitinib (Table 1). The transfusion status shift based on a rolling TI rate is presented in Supplemental Table S4A. In the overall population, fewer annual outpatient transfusion visits were recorded in the momelotinib vs. ruxolitinib arms in patients who were TD at baseline (9.39 vs. 16.17 visits) and TI/TR at baseline (0.63 vs. 3.82 visits) (Supplemental Table S5). 

The moderate-to-severe anemia subgroup included 86 patients in the momelotinib arm (58 with moderate anemia) and 94 patients in the ruxolitinib arm (73 with moderate anemia). While most patients with anemia in the momelotinib arm retained their baseline transfusion status at week 24, most patients with anemia who were TI/TR at baseline became TD at week 24 with ruxolitinib treatment (Table 1). The transfusion status shift for the moderate-to-severe anemia subgroup based on a rolling TI rate is presented in Supplemental Table S4B. Fewer annual outpatient transfusion visits were recorded in the momelotinib vs. ruxolitinib arms in patients with moderate-to-severe anemia and patients with moderate anemia who were TD at baseline (9.58 vs. 17.43 and 10.66 vs. 17.78 visits, respectively) and TI/TR at baseline (1.88 vs. 7.46 visits for both) (Supplemental Table S5).

The ≥65-year-old population included 125 patients in the momelotinib arm and 122 patients in the ruxolitinib arm. Similar to the overall population, most patients in both treatment arms retained their baseline transfusion status at week 24, and a greater proportion of patients were TD at week 24 with ruxolitinib (Table 1). In this subgroup, fewer annual outpatient transfusion visits were recorded in the momelotinib vs. ruxolitinib arms in patients who had baseline TD (10.27 vs. 17.87 visits) and baseline TI/TR (0.82 vs. 5.63 visits) (Supplemental Table S5).

#### 3.1.2. Projected Differences in Total Annual Medical Costs

**Figure 2:**
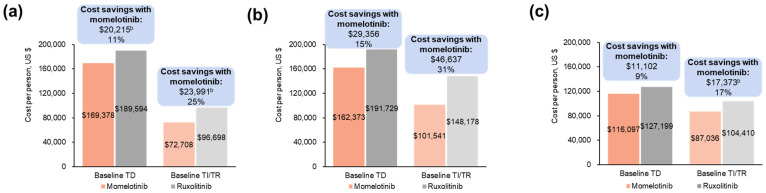
Projected annual medical costs by baseline transfusion status associated with momelotinib vs. ruxolitinib in the JAK inhibitor-naive population of SIMPLIFY-1 among (**a**) all patients ^a^, (**b**) patients with moderate-to-severe anemia ^a^, and (**c**) patients aged ≥65 years ^a^. TD, transfusion dependent; TI, transfusion independent; TR, transfusion requiring. ^a^ Each bar reflects costs for patients with a status of TD or TI/TR at week 24; costs are stratified by transfusion status at baseline. Savings are shown above the bars both as a USD amount and as a percentage of the higher cost. ^b^ Slight differences between base values and cost savings exist due to rounding.

Momelotinib was associated with projected cost savings compared with ruxolitinib in both TD and TI/TR patients across all populations evaluated. In the overall population, projected average annual total medical cost savings with momelotinib vs. ruxolitinib based on week 24 transfusion status were USD 20,215 per baseline TD patient and USD 23,991 per baseline TI/TR patient (Figure 2a; Supplemental Table S6A). In the moderate-to-severe anemia subgroup, projected average annual medical cost savings with momelotinib were USD 29,356 per baseline TD patient and USD 46,637 per baseline TI/TR patient (Figure 2b; Supplemental Table S6A). Results were similar in the moderate anemia subgroup, with projected average annual medical cost savings of USD 25,103 per baseline TD patient and USD 46,637 per baseline TI/TR patient (Supplemental Figure S1a).

Based on a rolling TI rate, the projected average annual total medical cost savings with momelotinib vs. ruxolitinib were USD 37,708 per patient with baseline TD status and USD 39,508 per patient with baseline TI/TR status. In the moderate-to-severe anemia subpopulation, the projected average annual total medical cost savings with momelotinib vs. ruxolitinib were USD 45,869 per patient with baseline TD status and USD 69,534 per patient with baseline TI/TR status (Supplemental Figure S2a).

In patients aged ≥65 years, projected annual medical cost savings with momelotinib were USD 11,102 per patient with baseline TD status and USD 17,373 per patient with baseline TI/TR status (Figure 2c; Supplemental Table S6A).

#### 3.1.3. Projected Differences in Annual Outpatient Transfusion Visit Costs

**Figure 3:**
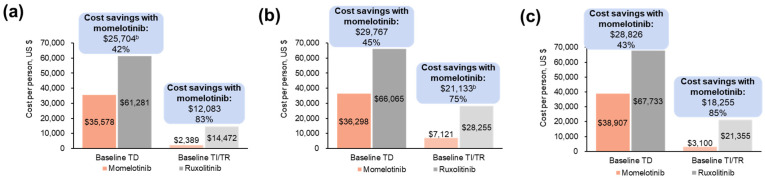
Projected annual outpatient transfusion visit costs by baseline transfusion status associated with momelotinib vs. ruxolitinib in the JAK inhibitor-naive population of SIMPLIFY-1 among (**a**) all patients ^a^, (**b**) patients with moderate-to-severe anemia ^a^, and (**c**) patients aged ≥65 years ^a^. TD, transfusion dependent; TI, transfusion independent; TR, transfusion requiring. ^a^ Each bar reflects costs for patients with a status of TD or TI/TR at week 24; costs are stratified by transfusion status at baseline. Savings are shown above the bars both as a USD amount and as a percentage of the higher cost. ^b^ Slight differences between base values and cost savings exist due to rounding.

Reductions in transfusions resulted in savings in projected outpatient transfusion costs with momelotinib vs. ruxolitinib in all populations. Fewer visits were associated with projected annual savings of USD 25,704 (baseline TD) and USD 12,083 (baseline TI/TR) with momelotinib (Figure 3a; Supplemental Table S6A). In the moderate-to-severe anemia subgroup, projected annual outpatient transfusion visit cost savings with momelotinib were USD 29,767 in patients with baseline TD and USD 21,133 in patients with baseline TI/TR (Figure 3b; Supplemental Table S6A); in the moderate anemia subgroup, these savings were USD 26,985 and USD 21,148, respectively (Supplemental Figure S1b). In patients aged ≥65 years, fewer visits were associated with projected annual outpatient transfusion visit cost savings with momelotinib of USD 28,826 in patients with baseline TD and USD 18,255 in patients with baseline TI/TR (Figure 3c; Supplemental Table S6A).

#### 3.1.4. Projected Differences in Annual Transfusion-Related Time Burden for Patients

**Figure 4:**
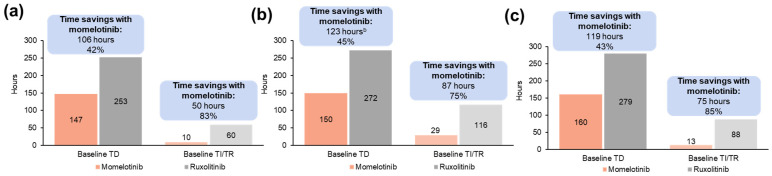
Projected annual transfusion-related time burden by baseline transfusion status associated with momelotinib vs. ruxolitinib in the JAK inhibitor-naive population of SIMPLIFY-1 among (**a**) all patients ^a^, (**b**) patients with moderate-to-severe anemia ^a^, and (**c**) patients aged ≥65 years ^a^. TD, transfusion dependent; TI, transfusion independent; TR, transfusion requiring. ^a^ Each bar reflects time burden for patients with a status of TD or TI/TR at week 24; time burdens are stratified by transfusion status at baseline. Savings are shown above the bars both as a number of hours and as a percentage of the higher time burden. ^b^ Slight differences between base values and time savings exist due to rounding.

Reductions in transfusions with momelotinib corresponded to reduced projected time burden for patients in all populations. Projected annual patient time savings in transfusion visits for momelotinib vs. ruxolitinib totaled 106 h per patient with baseline TD (147 vs. 253 h), which included 42 h of preparation/waiting, 50 h of transfusion/recovery, and 15 h of travel time saved (Supplemental Table S7A). Among patients who were TI/TR at baseline, the projected average annual savings in transfusion visit time associated with momelotinib vs. ruxolitinib totaled 50 h per patient (10 vs. 60 h) (Figure 4a; Supplemental Table S6A). In the moderate-to-severe anemia subgroup, 123 h were projected to be saved annually for transfusion visits for momelotinib vs. ruxolitinib per patient with baseline TD (150 vs. 272 h). Among patients who were TI/TR at baseline, the projected average annual savings in transfusion visit time associated with momelotinib vs. ruxolitinib totaled 87 h per patient (29 vs. 116 h) (Figure 4b; Supplemental Table S6A). Despite the lower transfusion burden in patients with moderate anemia, savings with momelotinib were maintained in that subgroup for both baseline TD (111 h; 167 vs. 278) and TI/TR (87 h; 29 vs. 117) patients (Supplemental Figure S1c).

In patients aged ≥65 years, 119 h were projected to be saved annually for transfusion visits for momelotinib vs. ruxolitinib per patient with baseline TD (160 vs. 279 h), which included 47 h of preparation/waiting, 56 h of transfusion/recovery, and 16 h of travel time saved (Supplemental Table S7B). Among patients who were TI/TR at baseline, the projected average annual savings in transfusion visit time associated with momelotinib vs. ruxolitinib totaled 75 h per patient (13 vs. 88 h) (Figure 4c; Supplemental Table S6A).

### 3.2. JAK Inhibitor-Experienced (SIMPLIFY-2)

#### 3.2.1. Transfusion Status and Rates

The JAK inhibitor-experienced population of SIMPLIFY-2 included 104 patients in the momelotinib arm vs. 52 patients in the BAT arm. Although most patients in both treatment arms retained their baseline transfusion status at week 24, more patients who were TI/TR at baseline became TD with BAT compared with momelotinib (Table 2). The transfusion status shift based on a rolling TI rate is presented in Supplemental Table S8A. Overall, there were fewer annual outpatient transfusion visits in the momelotinib vs. BAT arms in patients who had baseline TD (12.77 vs. 15.45 visits) and baseline TI/TR (3.5 vs. 5.04 visits) (Supplemental Table S9).

The moderate-to-severe anemia subgroup included 66 patients in the momelotinib arm and 39 patients in the BAT arm. Regardless of whether patients were TI/TR or TD at baseline, most patients in this anemic subpopulation became TD at week 24 (Table 2). Transfusion status shift based on a rolling TI rate is presented in Supplemental Table S8B. In the moderate-to-severe anemia subgroup, annual outpatient transfusion visits in the momelotinib vs. BAT arms were 13.24 vs. 15.73, respectively, in patients who were baseline TD and 7.45 vs. 5.59, respectively, in patients who were baseline TI/TR (Supplemental Table S9).

The ≥65-year-old population included 63 patients in the momelotinib arm vs. 38 patients in the BAT arm. Most patients in both arms retained their baseline transfusion status at week 24; however, there was a larger proportion of TD patients at week 24 in the BAT treatment arm (Table 2). In these patients, fewer annual outpatient transfusion visits were recorded in the momelotinib vs. BAT arms in patients who had baseline TD (13.35 vs. 16.59 visits) and baseline TI/TR (3.46 vs. 4.89 visits) (Supplemental Table S9).

#### 3.2.2. Projected Differences in Total Annual Medical Costs

**Figure 5:**
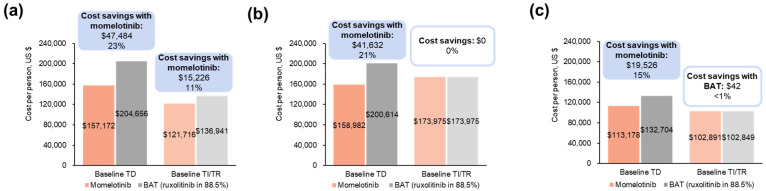
Projected annual medical costs (drug costs not included) by baseline transfusion status associated with momelotinib vs. BAT in the JAK inhibitor-experienced population of SIMPLIFY-2 among (**a**) all patients ^a^, (**b**) patients with moderate-to-severe anemia ^a^, and (**c**) patients aged ≥65 years ^a^. BAT, best available therapy; TD, transfusion dependent; TI, transfusion independent; TR, transfusion requiring. ^a^ Each bar reflects costs for patients with a status of TD or TI/TR at week 24; costs are stratified by transfusion status at baseline. Savings are shown above the bars both as a USD amount and as a percentage of the higher cost.

In the overall population, projected average annual total medical cost savings with momelotinib vs. BAT were USD 47,484 per baseline TD patient and USD 15,226 per baseline TI/TR patient (Figure 5a; Supplemental Table S6B). In the moderate-to-severe anemia subgroup, projected average annual medical cost savings with momelotinib were USD 41,632 per baseline TD patient. In patients with baseline TI/TR, there were no projected annual savings with momelotinib (Figure 5b; Supplemental Table S6B). 

Based on a rolling TI rate, the projected savings with momelotinib vs. BAT in the overall population were USD 60,989 per patient with baseline TD status and USD 58,136 per patient with baseline TI/TR status. In the moderate-to-severe anemia subpopulation, the projected savings with momelotinib vs. BAT were USD 55,801 per patient with baseline TD status and USD 32,486 per patient with baseline TI/TR status (Supplemental Figure S2b).

In patients aged ≥65 years, projected savings with momelotinib were USD 19,526 per baseline TD patient. In patients with baseline TI/TR, as in the anemic subgroup, the projected annual savings were USD 42 with BAT (Figure 5c; Supplemental Table S6B).

#### 3.2.3. Projected Differences in Annual Outpatient Transfusion Visit Costs

**Figure 6:**
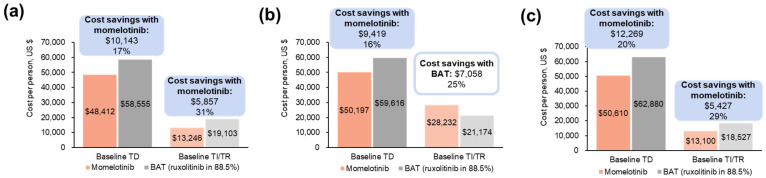
Projected annual outpatient transfusion visit costs by baseline transfusion status associated with momelotinib vs. BAT in the JAK inhibitor-experienced population of SIMPLIFY-2 among (**a**) all patients ^a^, (**b**) patients with moderate-to-severe anemia ^a^, and (**c**) patients aged ≥65 years ^a^. BAT, best available therapy; TD, transfusion dependent; TI, transfusion independent; TR, transfusion requiring. ^a^ Each bar reflects costs for patients with a status of TD or TI/TR at week 24; costs are stratified by transfusion status at baseline. Savings are shown above the bars both as a USD amount and as a percentage of the higher cost.

In patients who were TD at baseline, momelotinib was associated with reduced outpatient transfusion visit costs. In the overall population, fewer visits were associated with projected annual savings of USD 10,143 (baseline TD) and USD 5857 (baseline TI/TR) with momelotinib (Figure 6a; Supplemental Table S6B). Projected savings with momelotinib in the moderate-to-severe anemia subgroup were USD 9419 (baseline TD). Of the 13 patients with baseline TI/TR, savings of USD 7058 were instead experienced by patients treated with BAT (Figure 6b; Supplemental Table S6B). In patients aged ≥65 years, fewer visits were associated with projected savings with momelotinib vs. BAT of USD 12,269 in patients with baseline TD and USD 5427 in patients with baseline TI/TR (Figure 6c; Supplemental Table S6B).

#### 3.2.4. Projected Differences in Annual Transfusion-Related Time Burden for Patients

**Figure 7:**
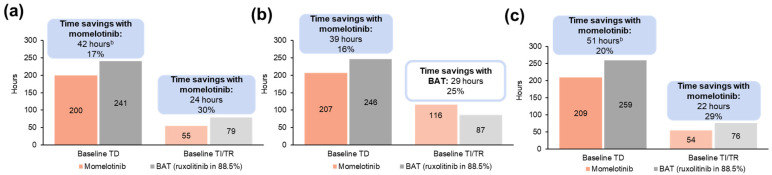
Projected annual transfusion-related time burden by baseline transfusion status associated with momelotinib vs. BAT in the JAK inhibitor-experienced population of SIMPLIFY-2 among (**a**) all patients ^a^, (**b**) patients with moderate-to-severe anemia ^a^, and (**c**) patients aged ≥65 years ^a^. BAT, best available therapy; TD, transfusion dependent; TI, transfusion independent; TR, transfusion requiring. ^a^ Each bar reflects costs for patients with a status of TD or TI/TR at week 24; costs are stratified by transfusion status at baseline. Savings are shown above the bars both as a number of hours and as a percentage of the higher time burden. ^b^ Slight differences between base values and time savings exist due to rounding.

In the overall population, projected annual patient time savings in transfusion visits for momelotinib vs. BAT totaled 42 h per patient with baseline TD (200 vs. 241 h), which included 16 h of preparation/waiting, 20 h of transfusion/recovery, and 6 h of travel time saved (Supplemental Table S10A). Among patients who were TI/TR at baseline, the projected savings associated with momelotinib vs. BAT totaled 24 h per patient (55 vs. 79 h) (Figure 7a; Supplemental Table S6B). In the moderate-to-severe anemia subgroup, 39 h were projected to be saved annually for transfusion visits for momelotinib vs. BAT per patient with baseline TD (207 vs. 246 h). Among patients who were TI/TR at baseline, the projected savings were associated with BAT and totaled 29 h per patient (116 vs. 87 h) (Figure 7b; Supplemental Table S6B). 

In patients aged ≥65 years, 51 h were projected to be saved annually for transfusion visits with momelotinib vs. BAT per patient with baseline TD (209 vs. 259 h), which included 20 h of preparation/waiting, 24 h of transfusion/recovery, and 7 h of travel time saved (Supplemental Table S10B). Among patients who were TI/TR at baseline, the projected savings associated with momelotinib vs. BAT totaled 22 h per patient (54 vs. 76 h) (Figure 7c; Supplemental Table S6B).

## 4. Discussion

This analysis showed that reductions in the need for transfusions associated with momelotinib are projected to result in meaningful healthcare system and patient savings in medical and transfusion-related costs relative to ruxolitinib in JAK inhibitor-naive patients and relative to BAT in JAK inhibitor-experienced patients with MF. Momelotinib was associated with projected cost and time savings in both TD and TI/TR patients across all populations evaluated in JAK inhibitor-naive and -experienced patients. The potential for cost savings with momelotinib treatment in TD patients is particularly relevant because TD patients experience a higher economic burden than TI/TR patients [11,12]. 

Anemia is associated with poor survival and QOL, negatively impacting patients’ social, emotional, and general health status, especially in patients who are symptomatic and require transfusions [4,8,24,25]. In this study, results showed that patients with anemia had higher projected medical and transfusion-related cost savings with momelotinib vs. ruxolitinib. These savings were observed in both the larger moderate-to-severe anemia subgroup (hemoglobin < 10 g/dL) and the moderate anemia subgroup (hemoglobin ≥ 8 to <10 g/dL), which is notable given the overall lower transfusion burden in the latter, suggesting that the benefits of momelotinib are not restricted to those with severe anemia and/or heavy transfusion burden. Progression to TD/TR status is also likely reduced in these groups in the momelotinib treatment arm relative to the ruxolitinib treatment arm; thus, potential cost savings with momelotinib relative to alternative treatments can come from both reducing transfusions and by reducing the proportion of patients who progress to TD. In JAK inhibitor-experienced patients, projected average annual medical cost savings with momelotinib were similar in the overall population and the moderate-to-severe anemia subgroup in patients who were TD at baseline. In patients who were TI/TR at baseline, there were no projected annual savings with momelotinib in the anemic subgroup or in patients aged ≥65 years due to the similar observed week 24 TD rates for momelotinib and BAT among these small subpopulations. 

Findings from the overall populations of the SIMPLIFY-1 and SIMPLIFY-2 studies were consistent in patients aged ≥65 years. Because the diagnosis of MF occurs most often between the ages of 65 and 70 years, the total costs of care for older patients are especially relevant. Advanced age also correlates with a poorer prognosis in MF [26]. Furthermore, the projected decrease in transfusion burden with momelotinib in patients in the overall population as well as those aged ≥65 years has the potential to improve patients’ QOL, which may be due in part to these reductions in transfusion-related burdens [27].

Projected annual outpatient transfusion visit cost savings and transfusion-related time burden savings with momelotinib were also similar in the overall population and the anemic subgroups in baseline TD patients in both SIMPLIFY-1 and SIMPLIFY-2, as well as in baseline TI/TR patients in SIMPLIFY-1. In baseline TI/TR patients in SIMPLIFY-2, transfusion visit cost savings of USD 7058 and time burden savings of 29 h were instead experienced by patients in the anemic subgroup treated with BAT, a result that should be interpreted with caution due to the small sample size (n = 13) in this subpopulation. Transfusion visit cost savings experienced by patients treated with BAT may be coming primarily from the inpatient setting, given the similarity in overall annual medical costs among baseline TI/TR patients in both treatment arms of this anemic subgroup. 

Due to limitations in data availability, this study used different definitions of transfusion status between data sources. While the definitions of TD used similar ratios (1 transfusion per 2 weeks), the time window over which transfusion dependence and transfusion independence were assessed differed between the SIMPLIFY trials and IBM MarketScan Commercial and Medicare Fee-for-Service analyses. Furthermore, patient populations also differed between data sources. While SIMPLIFY-1 included only JAK inhibitor-naive patients and SIMPLIFY-2 included only JAK inhibitor-experienced patients, estimates from the IBM MarketScan Commercial and Medicare Fee-for-Service analyses were based on both JAK inhibitor-naive and -experienced patients. This was not expected to change the direction of the estimated differences but could potentially affect the magnitude of change observed because the same costs are applied to both groups. Most patients in SIMPLIFY-2 were severely anemic, precluding an analysis of patients with moderate anemia in a JAK inhibitor-experienced setting. In contrast, the JAK inhibitor-naive setting of SIMPLIFY-1 enabled analysis of the moderate anemia subgroup, demonstrating that cost and time savings were observed even in this population with lower transfusion burden. Finally, time burden for transfusions was estimated using a previous analysis in patients with TD β-thalassemia because estimates for patients with MF were not available. Although transfusion visits for patients with β-thalassemia may not be directly comparable to those for patients with MF, as variability can exist with disease severity and clinical setting, they are expected to be similar in terms of the time burden required. It is important to note that the extrapolated data presented in this study are not precise numerical cost and time values but rather demonstrate the overall trend projected for reduced cost and time burden associated with momelotinib treatment.

## 5. Conclusions

This analysis demonstrates that reductions in the need for transfusions and TD status associated with momelotinib are projected to result in meaningful healthcare system and patient savings in medical and transfusion-related costs relative to ruxolitinib in JAK inhibitor-naive patients and relative to BAT in JAK inhibitor-experienced patients with MF. 

Findings were consistent in patients aged ≥65 years, patients with TD or TI/TR status at baseline, and patients with anemia, including those with moderate anemia. Among JAK inhibitor-experienced patients with MF, patients with anemia who were TD at baseline had projected medical and transfusion-related cost savings with momelotinib that were similar to the overall population but were not projected to experience cost savings if they were TI/TR at baseline. Momelotinib is estimated to provide time savings for patients on transfusion-related activities such as travel, RBC transfusion procedures and recovery, preparation, and waiting vs. ruxolitinib or BAT. Future studies of real-world data are warranted to further evaluate the impact of momelotinib on the clinical, economic, and humanistic burden in patients with MF. The present analyses were conducted prior to regulatory approval of momelotinib, and thus drug costs are not included. The offset between drug costs and potential transfusion-related cost savings will be an important future direction to explore and will complement current budget impact and cost effectiveness models developed to explore this question [28,29,30]. Overall, achieving or maintaining transfusion independence and maintaining hemoglobin levels in patients diagnosed with MF can minimize healthcare costs and reduce the financial burden for patients and healthcare systems at large. 

## Figures and Tables

**Table 1:** Transfusion status shift from baseline over the 24-week randomized treatment period in the phase 3 SIMPLIFY-1 trial.

			Week 24 Transfusion Status, n (%) ^a^		
		Baseline Transfusion Status	TI/TR	TD	Total
**Overall population**	Momelotinib	TI/TR	130 (80)	32 (20)	162 (75)
		TD	20 (38)	33 (62)	53 (25)
		Total	150 (70)	65 (30)	215
	Ruxolitinib	TI/TR	115 (70)	50 (30)	165 (76)
		TD	15 (29)	37 (71)	52 (24)
		Total	130 (60)	87 (40)	217
**Moderate-to-severe anemia** **subpopulation** **(Hb < 10 g/dL)**	Momelotinib	TI/TR	25 (68)	12 (32)	37 (43)
		TD	20 (41)	29 (59)	49 (57)
		Total	45 (52)	41 (48)	86
	Ruxolitinib	TI/TR	24 (47)	27 (53)	51 (54)
		TD	12 (28)	31 (72)	43 (46)
		Total	36 (38)	58 (62)	94
**Moderate anemia subpopulation** **(Hb ≥ 8 to <10 g/dL)**	Momelotinib	TI/TR	25 (68)	12 (32)	37 (64)
		TD	9 (43)	12 (57)	21 (36)
		Total	34 (59)	24 (41)	58
	Ruxolitinib	TI/TR	24 (47)	27 (53)	51 (70)
		TD	7 (33)	15 (68)	22 (30)
		Total	31 (42)	42 (58)	73
**Patients aged** **≥65 years**	Momelotinib	TI/TR	64 (75)	21 (25)	85 (68)
		TD	14 (35)	26 (65)	40 (32)
		Total	78 (62)	47 (38)	125
	Ruxolitinib	TI/TR	58 (64)	33 (36)	91 (75)
		TD	8 (26)	23 (74)	31 (25)
		Total	66 (54)	56 (46)	122

Hb, hemoglobin; TD, transfusion dependent; TI, transfusion independent; TR, transfusion requiring. ^a^ The percentage is the proportion of patients with the indicated week 24 transfusion status in each baseline transfusion stratification group or total population. Transfusion status was based on a TI rate at week 24 defined as a prespecified terminal 12-week definition of no transfusions and all hemoglobin levels of ≥8 g/dL in the last 12 weeks before week 24.

**Table 2:** Transfusion status shift from baseline over the 24-week randomized treatment period in the phase 3 SIMPLIFY-2 trial.

			Week 24 Transfusion Status, n (%) ^a^		
		Baseline Transfusion Status	TI/TR	TD	Total
**Overall population**	Momelotinib	TI/TR	27 (59)	19 (41)	46 (44)
		TD	25 (43)	33 (57)	58 (56)
		Total	52 (50)	52 (50)	104
	BAT	TI/TR	13 (52)	12 (48)	25 (48)
		TD	6 (22)	21 (78)	27 (52)
		Total	19 (37)	33 (63)	52
**Moderate-to-severe anemia** **subpopulation** **(Hb < 10 g/dL)**	Momelotinib	TI/TR	5 (36)	9 (64)	14 (21)
		TD	22 (42)	30 (58)	52 (79)
		Total	27 (41)	39 (59)	66
	BAT	TI/TR	5 (36)	9 (64)	14 (36)
		TD	6 (24)	19 (76)	25 (64)
		Total	11 (28)	28 (72)	39
**Patients aged** **≥65 years**	Momelotinib	TI/TR	12 (55)	10 (45)	22 (35)
		TD	19 (46)	22 (54)	41 (65)
		Total	31 (49)	32 (51)	63
	BAT	TI/TR	9 (56)	7 (44)	16 (42)
		TD	4 (18)	18 (82)	22 (58)
		Total	13 (34)	25 (66)	38

BAT, best available therapy; Hb, hemoglobin; TD, transfusion dependent; TI, transfusion independent; TR, transfusion requiring. ^a^ The percentage is the proportion of patients with the indicated week 24 transfusion status in each baseline transfusion stratification group or total population. Transfusion status was based on a TI rate at week 24 defined as a prespecified terminal 12-week definition of no transfusions and all hemoglobin levels of ≥8 g/dL in the last 12 weeks before week 24.

## Data Availability

Data are available upon reasonable request. Information on GSK’s data sharing commitments and requesting access to anonymized individual participant data and associated study documents can be found at https://www.gsk-studyregister.com/en/ (accessed on 3 December 2024).

## Supplementary Materials

The following supporting information can be downloaded at: https://www.mdpi.com/article/10.3390/cancers16234067/s1. Figure S1: Projected (a) total annual medical costs, (b) annual outpatient transfusion visit costs, and (c) transfusion-related time burden in the JAK inhibitor-naive population of SIMPLIFY-1 for patients in the moderate anemia subpopulation (hemoglobin ≥ 8 to <10 g/dL). Figure S2: Projected medical costs in the (a) JAK inhibitor-naive population of SIMPLIFY-1 and the (b) JAK inhibitor-experienced population of SIMPLIFY-2 using a rolling TI rate in the overall population and for patients with moderate-to-severe anemia (hemoglobin < 10 g/dL). Table S1: Criteria for transfusion status. Table S2: Costs of care from the IBM MarketScan Commercial and Medicare Fee-for-Service databases. Table S3: Patient time burden associated with RBC transfusions. Table S4: Transfusion status shift from baseline over the 24-week randomized treatment period in the phase 3 SIMPLIFY-1 trial (rolling TI definition). Table S5: Mean frequency of transfusion visits of patients enrolled in SIMPLIFY-1 over the course of a month and over the course of a year stratified by treatment arm and transfusion status at baseline. Table S6: Summary of projected transfusion-related cost and time savings with momelotinib. Table S7: Projected transfusion time savings per year and per month for momelotinib vs. ruxolitinib in SIMPLIFY-1. Table S8: Transfusion status shift from baseline over the 24-week randomized treatment period in the phase 3 SIMPLIFY-2 trial (rolling TI definition). Table S9: Mean frequency of transfusion visits of patients enrolled in SIMPLIFY-2 over the course of a month and over the course of a year stratified by treatment arm and transfusion status at baseline. Table S10: Projected transfusion time savings per year and per month for momelotinib vs. BAT in SIMPLIFY-2.
