# Supplementary material for: Transfusion-Related Cost and Time Burden Offsets in Patients with Myelofibrosis Treated with Momelotinib in the SIMPLIFY-1 and SIMPLIFY-2 Trials
Source: Cancers (Basel). 2024 Dec 5;16(23):4067. doi: 10.3390/cancers16234067 (PMC11640613; doi:10.3390/cancers16234067)
Supplement: Supplementary file 1 [file cancers-16-04067-s001.zip › cancers-3290022-supplementary.pdf]

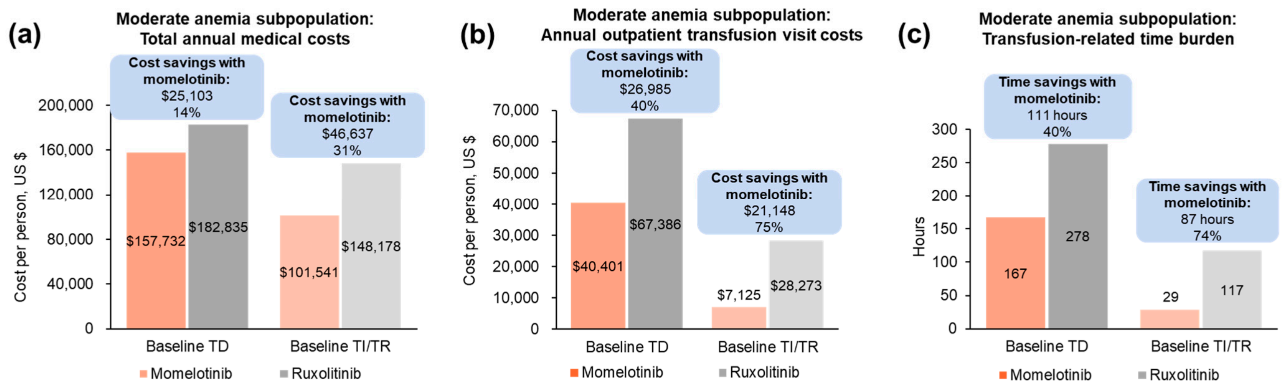


**Figure S1.** Projected (a) total annual medical costs, (b) annual outpatient transfusion visit costs, and (c) transfusion-related time burden in the JAK inhibitor–naive population of SIMPLIFY-1 for patients in the moderate anemia subpopulation (hemoglobin ≥8 to <10 g/dL)[a]

JAK, Janus kinase; TD, transfusion dependent; TI, transfusion independent; TR, transfusion requiring.

[a] Each bar reflects costs or time burdens for patients with a status of TD or TI/TR at week 24 (terminal TI definition); costs and time burdens are stratified by transfusion status at baseline. Savings are shown above the bars both as a US $ amount (for costs) or number of hours (for time burdens) and as a percentage of the higher cost or time burden.

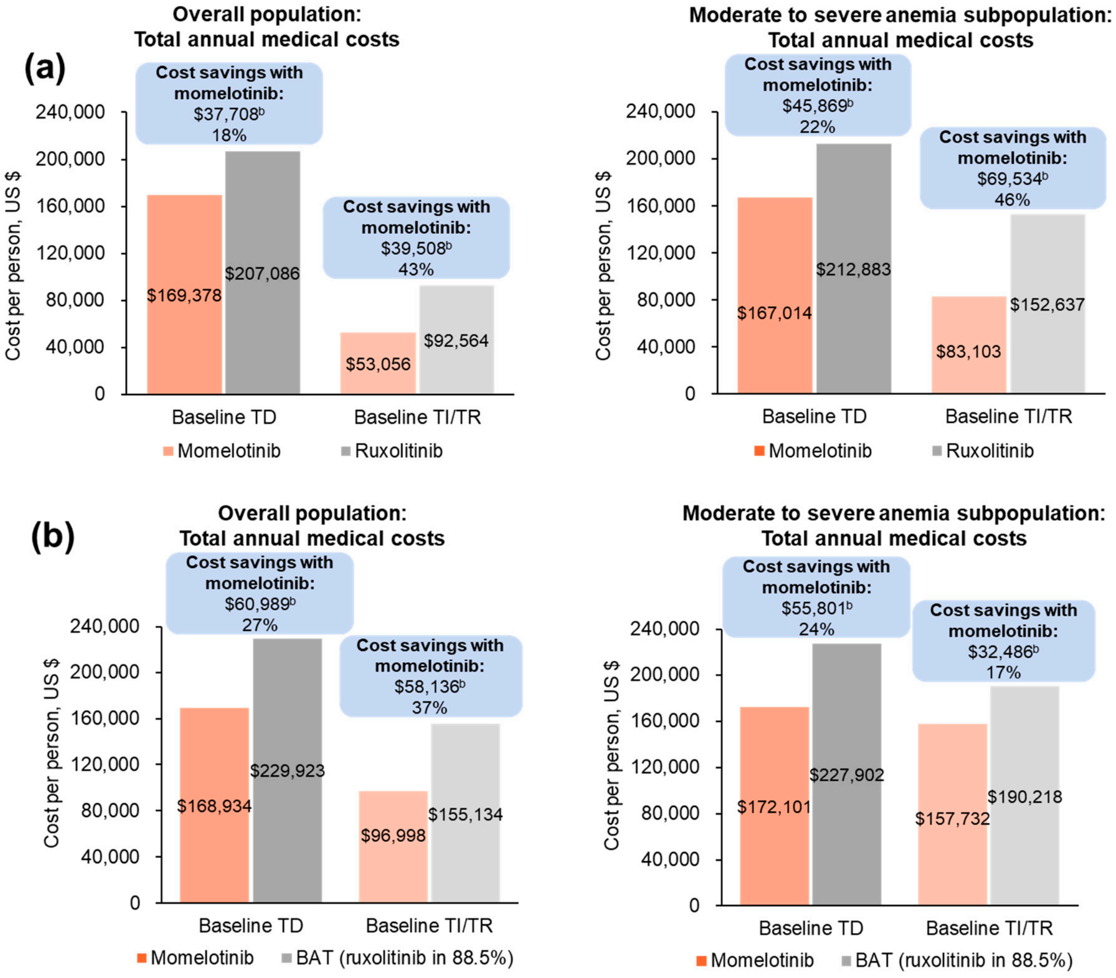


**Figure S2.** Projected medical costs in the (a) JAK inhibitor–naive population of SIMPLIFY-1 and the (b) JAK inhibitor–experienced population of SIMPLIFY-2 using a rolling TI rate in the overall population and for patients with moderate to severe anemia (hemoglobin <10 g/dL)[a]

BAT, best available therapy; JAK, Janus kinase; TD, transfusion dependent; TI, transfusion independent; TR, transfusion requiring.

[a] Costs were calculated from a rolling TI rate based on no transfusions and all hemoglobin levels of ≥8 g/dL during any 12-week period through week 24. Savings are shown above the bars both as a US $ amount and as a percentage of the higher cost.

[b] Differences in cost burden are calculated as cost burden for momelotinib minus cost burden for ruxolitinib in SIMPLIFY-1 and minus cost burden for BAT in SIMPLIFY-2. Differences of <0 indicate cost savings for momelotinib relative to ruxolitinib/BAT; differences of >0 indicate cost savings for ruxolitinib/BAT relative to momelotinib.

**Table S1.** Criteria for transfusion status[a]

| | SIMPLIFY-1 and SIMPLIFY-2[b] | IBM MarketScan Commercial analysis[c] | Medicare Fee-for-Service analysis |
|---|---|---|---|
| **TD** | ≥4 RBC units transfused or a hemoglobin level of <8 g/dL in the prior 8 weeks | ≥2 RBC transfusions within any 4-week period in the prior 2 years | ≥6 units of RBC transfusions or whole blood transfusions in any 12-week period |
| **TI** | Terminal TI rate: No RBC transfusions and all hemoglobin levels of ≥8 g/dL in the prior 12 weeks<br><br>or<br><br>Rolling TI rate: No transfusions and all hemoglobin levels of ≥8 g/dL during any 12-week period through week 24 | No RBC transfusions in the prior 2 years | No RBC transfusions during the 180 days following the earliest confirmed diagnosis of anemia |
| **TR** | Does not meet criteria for TD or TI | Does not meet criteria for TD or TI | Does not meet criteria for TD or TI |

RBC, red blood cell; TD, transfusion dependent; TI, transfusion independent; TR, transfusion requiring.

[a] Definitions were assumed as being functionally similar, as each study defines TI status as zero RBC transfusions and TD status as approximately 0.5-unit transfusions per week.
[b] SIMPLIFY-1 definitions of transfusion status from Mesa RA, et al. *J Clin Oncol.* 2017;35:3844-3850. SIMPLIFY-2 definitions of transfusion status from Harrison CN, et al. *Lancet Haematol*. 2018;5:e73-e81.
[c] IBM MarketScan Commercial analysis definitions of transfusion status from Gerds AT, et al. ASH 2022. Poster 1729.

**Table S2.** Costs of care from the IBM MarketScan Commercial and Medicare Fee-for-Service databases

| | **Cost estimates from the IBM MarketScan Commercial database[a]** | | | |
|---|---|---|---|---|
| **Outcome** | **Units** | **TI/TR[b]** | **TD[b]** | **All patients** |
| All-cause total medical costs | $/(person-year) | 27,789 | 225,190 | – |
| Transfusion visit[c] | $/outpatient transfusion claims | – | – | 3,790 |

| | **Cost estimates Medicare Fee-for-Service database[d]** | | | | |
|---|---|---|---|---|---|
| **Outcome** | **Units** | **TI[b]** | **TR[b]** | **TD[b]** | **All patients** |
| All-cause total medical costs | $/(person-year) | 69,961 | 133,561 | 136,047 | 98,223 |
| Transfusion visit[c] | $/outpatient transfusion claims | – | – | – | 3,790[e] |

JAK, Janus kinase; TD, transfusion dependent; TI, transfusion independent; TR, transfusion requiring.

[a] Due to availability in the IBM MarketScan Commercial database, estimates were based on JAK inhibitor–naive and –experienced patients.
[b] The IBM MarketScan Commercial study only reported cost data combined for the TI and TR groups.
[c] It was assumed that the mean cost per transfusion was equivalent to the mean cost for an outpatient transfusion claim.
[d] Due to availability in the Medicare Fee-for-Service database, estimates were available for JAK inhibitor–naive patients only. Costs for transfusion visits were not available from the Medicare Fee-for-Service database.
[e] It was, therefore, assumed that the mean cost per transfusion for Medicare was the same as in the IBM MarketScan Commercial database.

**Table S3.** Patient time burden associated with RBC transfusions[a]

| Outcomes | Average time spent per 1 RBC transfusion procedure, hours | |
|---|---|---|
| | **Mean** | **SD** |
| Preparation | 1.12 | 2.69 |
| Waiting room | 1.43 | 5.88 |
| Waiting for blood | 3.57 | 7.60 |
| RBC transfusion procedure and recovery | 7.36 | 11.26 |
| Travel | 2.14 | 4.16 |
| Total time | 15.62 | 23.24 |

[a] The patient population from the 2022 Knoth et al study consisted of those with β-thalassemia.

**Table S4.** Transfusion status shift from baseline over the 24-week randomized treatment period in the phase 3 SIMPLIFY-1 trial (rolling TI definition)

**A. Patients in the Overall Population–Rolling TI rate**

| | | **Week 24 transfusion status (rolling TI rate), n (%)[a]** | | |
|---|---|---|---|---|
| | **Baseline transfusion status** | **TI** | **Non-TI** | **Total** |
| **Momelotinib** | | | | |
| | **TI/TR** | 144 (89) | 18 (11) | **162 (75)** |
| | **TD** | 20 (38) | 33 (62) | **53 (25)** |
| | **Total** | 164 (76) | 51 (24) | **215** |
| **Ruxolitinib** | | | | |
| | **TI/TR** | 118 (72) | 47 (28) | **165 (76)** |
| | **TD** | 11 (21) | 41 (79) | **52 (24)** |
| | **Total** | 129 (59) | 88 (41) | **217** |

**B. Patients in the Moderate to Severe Anemia Subpopulation (hemoglobin <10 g/dL) – Rolling TI rate**

| | | Week 24 transfusion status (rolling TI rate), n (%)[a] | | |
|---|---|---|---|---|
| | **Baseline transfusion status** | **TI** | **Non-TI** | **Total** |
| **Momelotinib** | | | | |
| | **TI/TR** | 28 (76) | 9 (24) | **37 (43)** |
| | **TD** | 19 (39) | 30 (61) | **49 (57)** |
| | **Total** | 47 (55) | 39 (45) | **86** |
| **Ruxolitinib** | | | | |
| | **TI/TR** | 23 (45) | 28 (55) | **51 (54)** |
| | **TD** | 8 (19) | 35 (81) | **43 (46)** |
| | **Total** | 31 (33) | 63 (67) | **94** |

TD, transfusion dependent; TI, transfusion independent; TR, transfusion requiring.[a] Transfusion status was based on a rolling TI rate defined as no transfusions and all hemoglobin levels of ≥8 g/dL during any 12-week period through week 24.

**Table S5.** Mean frequency of transfusion visits of patients enrolled in SIMPLIFY-1 over the course of a month and over the course of a year in different subpopulations stratified by treatment arm and transfusion status at baseline[a]

| | Transfusion visits by subpopulation (mean) | | | | | | | |
|---|---|---|---|---|---|---|---|---|
| | Overall[b] | | Moderate to Severe Anemia (Hb <10 g/dL) | | Moderate Anemia (Hb ≥8 to <10 g/dL) | | Aged ≥65[c] | |
| **Treatment arm** | **Per month** | **Per year** | **Per month** | **Per year** | **Per month** | **Per year** | **Per month** | **Per year** |
| **Momelotinib (all patients)** | 0.23 | 2.79 | 0.52 | 6.27 | 0.42 | 5.06 | 0.32 | 3.84 |
| Baseline TI/TR | 0.05 | 0.63 | 0.16 | 1.88 | 0.16 | 1.88 | 0.07 | 0.82 |
| Baseline TD | 0.78 | 9.39 | 0.80 | 9.58 | 0.89 | 10.66 | 0.86 | 10.27 |
| **Ruxolitinib (all patients)** | 0.56 | 6.78 | 1.00 | 12.02 | 0.88 | 10.57 | 0.73 | 8.74 |
| Baseline TI/TR | 0.32 | 3.82 | 0.62 | 7.46 | 0.62 | 7.46 | 0.47 | 5.63 |
| Baseline TD | 1.35 | 16.17 | 1.45 | 17.43 | 1.48 | 17.78 | 1.49 | 17.87 |

Hb, hemoglobin; RBC, red blood cell; TD, transfusion dependent; TI, transfusion independent; TR, transfusion requiring.

[a] SIMPLIFY-1 defined TI as zero RBC transfusions and a hemoglobin level of ≥8 g/dL in the last 12 weeks of a 24-week period, TD as ≥4 RBC transfusions in the last 8 weeks of a 24-week period or hemoglobin levels of <8 g/dL, and TR as patients who were neither TI nor TD. Refer to **Supplemental Table S1**.

[b] Mean transfusion visits are calculated among the 432 patients with transfusion status data available at week 24.

[c] Mean transfusion visits are calculated among the 247 patients aged ≥65 years with transfusion status data available at week 24.

**Table S6.** Summary of projected transfusion-related cost and time savings with momelotinib

**A. JAK inhibitor–naive population of SIMPLIFY-1 (momelotinib vs ruxolitinib)**

| | **Baseline TD** | **Baseline TI/TR** |
|---|---|---|
| **All patients** | | |
| Medical costs | $20,215 | $23,991 |
| Outpatient transfusion visit costs | $25,704 | $12,083 |
| Transfusion-related time burden | 106 hours | 50 hours |
| **Moderate to severe anemia** | | |
| Medical costs | $29,356 | $46,637 |
| Outpatient transfusion visit costs | $29,767 | $21,133 |
| Transfusion-related time burden | 123 hours | 87 hours |
| **Age ≥65** | | |
| Medical costs | $11,102 | $17,373 |
| Outpatient transfusion visit costs | $28,826 | $18,255 |
| Transfusion-related time burden | 119 hours | 75 hours |

**B. JAK inhibitor–experienced population of SIMPLIFY-2 (momelotinib vs BAT)**

| | **Baseline TD** | **Baseline TI/TR** |
|---|---|---|
| **All patients** | | |
| Medical costs | $47,484 | $15,226 |
| Outpatient transfusion visit costs | $10,143 | $5,857 |
| Transfusion-related time burden | 42 hours | 24 hours |
| **Moderate to severe anemia** | | |
| Medical costs | $41,632 | $0 |
| Outpatient transfusion visit costs | $9,419 | -$7,058 |
| Transfusion-related time burden | 39 hours | -29 hours |
| **Age ≥65** | | |
| Medical costs | $19,526 | -$42 |
| Outpatient transfusion visit costs | $12,269 | $5,427 |
| Transfusion-related time burden | 51 hours | 22 hours |

BAT, best available therapy; TD, transfusion dependent; TI, transfusion independent; TR, transfusion requiring.

**Table S7.** Projected transfusion time savings per year and per month for momelotinib vs ruxolitinib in SIMPLIFY-1[a]

## A. Patients in the Overall Population

| | **Momelotinib treatment arm** | | | **Ruxolitinib treatment arm** | | | **Time savings (ruxolitinib – momelotinib)[b]** | | |
|---|---|---|---|---|---|---|---|---|---|
| | TI/TR status | TD status | All patients | TI/TR status | TD status | All patients | TI/TR status | TD status | Total |
| **Total visit time (hours/year)** | **9.85** | **146.63** | **43.56** | **59.65** | **252.56** | **105.88** | **49.80** | **105.93** | **62.31** |
| Preparation | 0.71 | 10.51 | 3.12 | 4.28 | 18.11 | 7.59 | 3.57 | 7.60 | 4.47 |
| Waiting room | 0.90 | 13.42 | 3.99 | 5.46 | 23.12 | 9.69 | 4.56 | 9.70 | 5.70 |
| Waiting for blood | 2.25 | 33.51 | 9.96 | 13.63 | 57.72 | 24.20 | 11.38 | 24.21 | 14.24 |
| RBCT procedure and recovery | 4.64 | 69.09 | 20.53 | 28.10 | 119.01 | 49.89 | 23.47 | 49.92 | 29.36 |
| Travel time | 1.35 | 20.09 | 5.97 | 8.17 | 34.60 | 14.51 | 6.82 | 14.51 | 8.54 |
| | | | | | | | | | |
| | **Momelotinib treatment arm** | | | **Ruxolitinib treatment arm** | | | **Time savings (ruxolitinib – momelotinib)[b]** | | |
| | TI/TR status | TD status | All patients | TI/TR status | TD status | All patients | TI/TR status | TD status | Total |
| **Total visit time (hours/month)** | **0.82** | **12.22** | **3.63** | **4.97** | **21.05** | **8.82** | **4.15** | **8.83** | **5.19** |
| Preparation | 0.06 | 0.88 | 0.26 | 0.36 | 1.51 | 0.63 | 0.30 | 0.63 | 0.37 |
| Waiting room | 0.08 | 1.12 | 0.33 | 0.46 | 1.93 | 0.81 | 0.38 | 0.81 | 0.48 |
| Waiting for blood | 0.19 | 2.79 | 0.83 | 1.14 | 4.81 | 2.02 | 0.95 | 2.02 | 1.19 |
| RBCT procedure and recovery | 0.39 | 5.76 | 1.71 | 2.34 | 9.92 | 4.16 | 1.96 | 4.16 | 2.45 |
| Travel time | 0.11 | 1.67 | 0.50 | 0.68 | 2.88 | 1.21 | 0.57 | 1.21 | 0.71 |

## B. Patients Aged ≥65 Years

| | **Momelotinib treatment arm** | | | **Ruxolitinib treatment arm** | | | **Time savings (ruxolitinib – momelotinib)[b]** | | |
|---|---|---|---|---|---|---|---|---|---|
| | TI/TR status | TD status | All patients | TI/TR status | TD status | All patients | TI/TR status | TD status | Total |
| **Total visit time (hours/year)** | **12.78** | **160.35** | **60.00** | **88.01** | **279.15** | **136.58** | **75.23** | **118.80** | **76.58** |
| Preparation | 0.92 | 11.50 | 4.30 | 6.31 | 20.02 | 9.79 | 5.39 | 8.52 | 5.49 |
| Waiting room | 1.17 | 14.68 | 5.49 | 8.06 | 25.56 | 12.50 | 6.89 | 10.88 | 7.01 |
| Waiting for blood | 2.92 | 36.65 | 13.71 | 20.12 | 63.80 | 31.22 | 17.20 | 27.15 | 17.51 |
| RBCT procedure and recovery | 6.02 | 75.55 | 28.27 | 41.47 | 131.53 | 64.36 | 35.45 | 55.98 | 36.09 |
| Travel time | 1.75 | 21.97 | 8.22 | 12.06 | 38.25 | 18.71 | 10.31 | 16.28 | 10.49 |
| | | | | | | | | | |
| | **Momelotinib treatment arm** | | | **Ruxolitinib treatment arm** | | | **Time savings (ruxolitinib – momelotinib)[b]** | | |
| | TI/TR status | TD status | All patients | TI/TR status | TD status | All patients | TI/TR status | TD status | Total |
| **Total visit time (hours/month)** | **1.06** | **13.36** | **5.00** | **7.33** | **23.26** | **11.38** | **6.27** | **9.90** | **6.38** |
| Preparation | 0.08 | 0.96 | 0.36 | 0.53 | 1.67 | 0.82 | 0.45 | 0.71 | 0.46 |
| Waiting room | 0.10 | 1.22 | 0.46 | 0.67 | 2.13 | 1.04 | 0.57 | 0.91 | 0.58 |
| Waiting for blood | 0.24 | 3.05 | 1.14 | 1.68 | 5.32 | 2.60 | 1.44 | 2.27 | 1.46 |
| RBCT procedure and recovery | 0.50 | 6.30 | 2.36 | 3.46 | 10.96 | 5.36 | 2.96 | 4.66 | 3.00 |
| Travel time | 0.15 | 1.83 | 0.69 | 1.00 | 3.19 | 1.56 | 0.85 | 1.36 | 0.87 |

EHA, European Hematology Association; RBCT, red blood cell transfusion; TD, transfusion dependent; TI, transfusion independent; TR, transfusion requiring.

[a] Costs of care were extracted from the 2022 Knoth et al study in their EHA poster submission. Estimate for 1 RBCT procedure was 15.62 mean hours/person-visit, which was stratified by time spent during procedure preparations (1.12), waiting room (1.43), waiting for blood (3.57), RBCT procedure and recovery (7.36), and round-trip travel time (2.14). Refer to **Supplemental Table S3**.
[b] Differences in time burden are calculated as time burden for ruxolitinib minus time burden for momelotinib. Differences of >0 indicate time savings for momelotinib relative to ruxolitinib; differences of <0 indicate time savings for ruxolitinib relative to momelotinib.

**Table S8.** Transfusion status shift from baseline over the 24-week randomized treatment period in the phase 3 SIMPLIFY-2 trial (rolling TI definition)

**A. Patients in the Overall Population–Rolling TI rate**

| | | Week 24 transfusion status (rolling TI rate), n (%)[a] | | |
|---|---|---|---|---|
| | **Baseline transfusion status** | **TI** | **Non-TI** | **Total** |
| **Momelotinib** | | | | |
| | **TI/TR** | 32 (70) | 14 (30) | **46 (44)** |
| | **TD** | 22 (38) | 36 (62) | **58 (56)** |
| | **Total** | 54 (52) | 50 (48) | **104** |
| **BAT** | | | | |
| | **TI/TR** | 11 (44) | 14 (56) | **25 (48)** |
| | **TD** | 3 (11) | 24 (89) | **27 (52)** |
| | **Total** | 14 (27) | 38 (73) | **52** |

**B. Patients in the Moderate to Severe Anemia Subpopulation (hemoglobin <10 g/dL) – Rolling TI rate**

| | | Week 24 transfusion status (rolling TI rate), n (%)[a] | | |
|---|---|---|---|---|
| | **Baseline transfusion status** | **TI** | **Non-TI** | **Total** |
| **Momelotinib** | | | | |
| | **TI/TR** | 6 (43) | 8 (57) | **14 (21)** |
| | **TD** | 19 (37) | 33 (63) | **52 (79)** |
| | **Total** | 25 (38) | 41 (62) | **66** |
| **BAT** | | | | |
| | **TI/TR** | 4 (29) | 10 (71) | **14 (36)** |
| | **TD** | 3 (12) | 22 (88) | **25 (64)** |
| | **Total** | 7 (18) | 32 (82) | **39** |

BAT, best available therapy; TD, transfusion dependent; TI, transfusion independent; TR, transfusion requiring.[a] Transfusion status was based on a rolling TI rate defined as no transfusions and all hemoglobin levels of ≥8 g/dL during any 12-week period through week 24.

**Table S9.** Mean frequency of transfusion visits of patients enrolled in SIMPLIFY-2 over the course of a month and over the course of a year in different subpopulations stratified by treatment arm and transfusion status at baseline[a]

| | Transfusion visits by subpopulation (mean) | | | | | |
|---|---|---|---|---|---|---|
| | **Overall[b]** | | **Moderate to Severe Anemia (Hb <10 g/dL)** | | **Aged ≥65[c]** | |
| **Treatment arm** | **Per month** | **Per year** | **Per month** | **Per year** | **Per month** | **Per year** |
| **Momelotinib (all patients)** | 0.72 | 8.67 | 1.00 | 12.02 | 0.82 | 9.90 |
| Baseline TI/TR | 0.29 | 3.50 | 0.62 | 7.45 | 0.29 | 3.46 |
| Baseline TD | 1.06 | 12.77 | 1.10 | 13.24 | 1.11 | 13.35 |
| **Ruxolitinib (all patients)** | 0.87 | 10.45 | 1.01 | 12.09 | 0.97 | 11.66 |
| Baseline TI/TR | 0.42 | 5.04 | 0.47 | 5.59 | 0.41 | 4.89 |
| Baseline TD | 1.29 | 15.45 | 1.31 | 15.73 | 1.38 | 16.59 |

BAT, best available therapy; Hb, hemoglobin; RBC, red blood cell; TD, transfusion dependent; TI, transfusion independent; TR, transfusion requiring.

[a] SIMPLIFY-2 defined TI as zero RBC transfusions and a hemoglobin level of ≥8 g/dL in the last 12 weeks of a 24-week period, TD as ≥4 RBC transfusions in the last 8 weeks of a 24-week period or hemoglobin levels of <8 g/dL, and TR as patients who were neither TI nor TD. Refer to **Supplemental Table S1**.

[b] Mean transfusion visits are calculated among the 156 patients with transfusion status data available at week 24.

[c] Mean transfusion visits are calculated among the 101 patients aged ≥65 years with transfusion status data available at week 24.

**Table S10.** Projected transfusion time savings per year and per month for momelotinib vs ruxolitinib in SIMPLIFY-2[a]

## A. Patients in the Overall Population

| | **Momelotinib treatment arm** | | | **BAT treatment arm** | | | **Time savings (BAT – momelotinib)[b]** | | |
|---|---|---|---|---|---|---|---|---|---|
| | TI/TR status | TD status | All patients | TI/TR status | TD status | All patients | TI/TR status | TD status | Total |
| **Total visit time (hours/year)** | **54.59** | **199.52** | **135.42** | **78.73** | **241.32** | **163.16** | **24.14** | **41.80** | **27.74** |
| Preparation | 3.91 | 14.31 | 9.71 | 5.65 | 17.30 | 11.70 | 1.73 | 3.00 | 1.99 |
| Waiting room | 5.00 | 18.27 | 12.40 | 7.21 | 22.09 | 14.94 | 2.21 | 3.83 | 2.54 |
| Waiting for blood | 12.48 | 45.60 | 30.95 | 17.99 | 55.16 | 37.29 | 5.52 | 9.55 | 6.34 |
| RBCT procedure and recovery | 25.72 | 94.01 | 63.81 | 37.10 | 113.71 | 76.88 | 11.37 | 19.70 | 13.07 |
| Travel time | 7.48 | 27.34 | 18.55 | 10.79 | 33.06 | 22.35 | 3.31 | 5.73 | 3.80 |
| | | | | | | | | | |
| | **Momelotinib treatment arm** | | | **BAT treatment arm** | | | **Time Savings (BAT – momelotinib)[b]** | | |
| | TI/TR status | TD status | All patients | TI/TR status | TD status | All patients | TI/TR status | TD status | Total |
| **Total visit time (hours/month)** | **4.55** | **16.63** | **11.28** | **6.56** | **20.11** | **13.60** | **2.01** | **3.48** | **2.31** |
| Preparation | 0.33 | 1.19 | 0.81 | 0.47 | 1.44 | 0.97 | 0.14 | 0.25 | 0.17 |
| Waiting room | 0.42 | 1.52 | 1.03 | 0.60 | 1.84 | 1.24 | 0.18 | 0.32 | 0.21 |
| Waiting for blood | 1.04 | 3.80 | 2.58 | 1.50 | 4.60 | 3.11 | 0.46 | 0.80 | 0.53 |
| RBCT procedure and recovery | 2.14 | 7.83 | 5.32 | 3.09 | 9.48 | 6.41 | 0.95 | 1.64 | 1.09 |
| Travel time | 0.62 | 2.28 | 1.55 | 0.90 | 2.76 | 1.86 | 0.28 | 0.48 | 0.32 |

## B. Patients Aged ≥65 Years

| | Momelotinib treatment arm | | | BAT treatment arm | | | Time savings (BAT – momelotinib)[b] | | |
|---|---|---|---|---|---|---|---|---|---|
| | TI/TR status | TD status | All patients | TI/TR status | TD status | All patients | TI/TR status | TD status | Total |
| **Total visit time (hours/year)** | **53.99** | **208.58** | **154.60** | **76.36** | **259.15** | **182.18** | **22.37** | **50.57** | **27.58** |
| Preparation | 3.87 | 14.96 | 11.09 | 5.48 | 18.58 | 13.06 | 1.61 | 3.62 | 1.97 |
| Waiting room | 4.94 | 19.10 | 14.15 | 6.99 | 23.73 | 16.68 | 2.05 | 4.63 | 2.53 |
| Waiting for blood | 12.34 | 47.67 | 35.33 | 17.45 | 59.23 | 41.64 | 5.11 | 11.56 | 6.31 |
| RBCT procedure and recovery | 25.44 | 98.28 | 72.85 | 35.98 | 122.11 | 85.84 | 10.54 | 23.83 | 12.99 |
| Travel time | 7.40 | 28.58 | 21.18 | 10.46 | 35.50 | 24.96 | 3.06 | 6.92 | 3.78 |
| | | | | | | | | | |
| | **Momelotinib treatment arm** | | | **BAT treatment arm** | | | **Time savings (BAT – momelotinib)[b]** | | |
| | TI/TR status | TD status | All patients | TI/TR status | TD status | All patients | TI/TR status | TD status | Total |
| **Total visit time (hours/month)** | **4.50** | **17.38** | **12.88** | **6.36** | **21.60** | **15.18** | **1.86** | **4.22** | **2.30** |
| Preparation | 0.32 | 1.25 | 0.92 | 0.46 | 1.55 | 1.09 | 0.14 | 0.30 | 0.17 |
| Waiting room | 0.41 | 1.59 | 1.18 | 0.58 | 1.98 | 1.39 | 0.17 | 0.39 | 0.21 |
| Waiting for blood | 1.03 | 3.97 | 2.94 | 1.45 | 4.94 | 3.47 | 0.42 | 0.97 | 0.53 |
| RBCT procedure and recovery | 2.12 | 8.19 | 6.07 | 3.00 | 10.18 | 7.15 | 0.88 | 1.99 | 1.08 |
| Travel time | 0.62 | 2.38 | 1.77 | 0.87 | 2.96 | 2.08 | 0.25 | 0.58 | 0.31 |

BAT, best available therapy; EHA, European Hematology Association; RBCT, red blood cell transfusion; TD, transfusion dependent; TI, transfusion independent; TR, transfusion requiring.

[a] Costs of care were extracted from the 2022 Knoth et al study in their EHA poster submission. Estimate for 1 RBCT procedure was 15.62 mean hours/person-visit, which was stratified by time spent during procedure preparations (1.12), waiting room (1.43), waiting for blood (3.57), RBCT procedure and recovery (7.36), and round-trip travel time (2.14). Refer to **Supplemental Table S3**.
[b] Differences in time burden are calculated as time burden for BAT minus time burden for momelotinib. Differences of >0 indicate time savings for momelotinib relative to BAT; differences of <0 indicate time savings for BAT relative to momelotinib.
